# Selective Anticancer Effects of a P-I Metalloproteinase from Bothrops Moojeni Snake Venom (BthMP) on Lung Cancer Cells

**DOI:** 10.3390/ph19030428

**Published:** 2026-03-06

**Authors:** Vinícius Queiroz Oliveira, Luísa Carregosa Santos, Leonardo Oliveira Silva Bastos Andrade, Lucas Miranda Marques, Amélia Cristina Mendes de Magalhães Gusmão, Thiago Macedo Lopes Correia, Samuel Cota Teixeira, Eloisa Amália Vieira Ferro, Veridiana de Melo Rodrigues, Sarah Natalie Cirilo Gimenes, Mônica Colombini, Patricia Bianca Clissa, Sabri Saeed Sanabani, Daiana Silva Lopes

**Affiliations:** 1 Institute Multidisciplinary in Health, Universidade Federal da Bahia (UFBA), Vitória da Conquista 45029-094, BA, Brazil; viniciusqueirozbio@gmail.com (V.Q.O.); luisacarregosabio@gmail.com (L.C.S.); oliveiraleonardo997@gmail.com (L.O.S.B.A.); lmirandamarques@gmail.com (L.M.M.); ameliavix@hotmail.com (A.C.M.d.M.G.); 2 Laboratory of Biochemistry and Animal Toxins, Institute of Biotechnology, Universidade Federal de Uberlândia (UFU), Uberlândia 38405-318, MG, Brazil; veridiana@ufu.br; 3 Institute of Health Sciences, Universidade Federal da Bahia (UFBA), Salvador 40231-300, BA, Brazil; thiagomlc@ufba.br; 4 Laboratory of Immunophysiology of Reproduction, Institute of Biomedical Sciences, Universidade Federal de Uberlândia (UFU), Uberlândia 38405-318, MG, Brazil; samuel.teixeira@ufu.br (S.C.T.); eloisa.ferro@ufu.br (E.A.V.F.); 5 Laboratory of Immunopathology, Instituto Butantan, São Paulo 05585-000, SP, Brazil; sarah_gi_menes@hotmail.com (S.N.C.G.); monica.colombini@butantan.gov.br (M.C.); patricia.clissa@butantan.gov.br (P.B.C.); 6 Laboratory of Medical Investigation in Dermatology and Immunodeficiency, São Paulo Institute of Tropical Medicine, School of Medicine, Universidade de São Paulo (USP), São Paulo 05403-000, SP, Brazil; sabyem_63@yahoo.com

**Keywords:** antimetastatic, oxidative stress, A549 cells, cell adhesion, *Bothrops moojeni* svMP

## Abstract

**Background:** Lung cancer remains a leading cause of mortality, mainly due to aggressive metastasis and therapeutic resistance. Snake venom metalloproteinases (svMPs), particularly the P-I class, are promising sources for novel antitumor agents. **Objectives:** This study investigated the impacts of BthMP, a P-I svMPs from *Bothrops moojeni* venom, on human lung carcinoma (A549) cells in comparison to non-cancerous human bronchial epithelial cells (BEAS-2B). **Methods and Results:** BthMP demonstrated potent and selective anti-cancer activity. It significantly inhibited key metastatic processes in A549 cells, including adhesion, migration, and invasion, while suppressing long-term proliferation, as shown by reduced colony formation and increased lactate dehydrogenase (LDH) release. Mechanistically, BthMP induced a massive increase in intracellular reactive oxygen species (ROS) by over 2000% and elevated nitric oxide (NO) by 35% in A549 cells, driving a state of lethal oxidative stress. Crucially, these cytotoxic and anti-metastatic effects were minimal in BEAS-2B cells; BthMP even suppressed basal ROS and NO levels in this non-cancerous line. The anti-migratory effects of BthMP were completely dependent on its zinc-based catalytic activity, as they were abolished by pretreatment with ethylenediaminetetraacetic acid. By simultaneously disrupting cell–matrix interactions and inducing selective, catastrophic oxidative stress in cancer cells, BthMP presents a dual-pronged anti-metastatic mechanism. **Conclusions:** These findings establish BthMP as a promising therapeutic scaffold for developing novel treatments against lung cancer progression.

## 1. Introduction

Cancer is a leading cause of death worldwide and represents a significant global health burden. Among all malignancies, lung cancer is a principal driver of cancer-related mortality, with non-small cell lung cancer (NSCLC), particularly adenocarcinoma, representing a major clinical challenge due to its aggressive metastatic behavior and resistance to conventional therapies [1,2]. Metastasis, driven by processes such as cell migration, invasion, and adhesion, is fueled by dysregulated extracellular matrix (ECM) interactions and genetic heterogeneity, necessitating innovative approaches to target these pathways [3]. Current treatments, including chemotherapy and targeted therapies, often fail to address metastatic progression effectively, highlighting the need for new therapeutic strategies [4].

Snake venom metalloproteinases (svMPs) have garnered attention as potential antitumor agents due to their ability to disrupt ECM components and modulate cellular processes critical to cancer progression [5,6]. Studies on svMPs, such as jararhagin, a svMP from *Bothrops jararaca*, have demonstrated their capacity to inhibit cancer cell proliferation and metastatic behaviors in breast and melanoma models, suggesting potential applications in lung cancer [7]. P-I class svMPs, characterized by a single metalloproteinase domain and zinc-dependent catalytic activity, are notable for their low hemorrhagic activity compared to P-III svMPs, which reduces unintended vascular damage [8]. However, their specific effects on lung cancer cells, particularly through mechanisms like oxidative stress and selective cytotoxicity, remain underexplored.

BthMP, a P-I metalloproteinase from *Bothrops moojeni* venom, exhibits weak hemorrhagic activity and potent ECM degradation capabilities, making it a candidate for cancer therapy [9,10]. Its classification as a zinc-dependent metalloproteinase is established by the complete inhibition of its proteolytic activity by metal chelators, which confirms the essential role of a zinc atom in its catalytic site. Its potential to selectively target cancer cells, such as human lung carcinoma (A549), while sparing non-cancerous cells like human bronchial epithelial cells (BEAS-2B), positions it as a novel agent for lung cancer research.

This study aims to evaluate the antitumoral effects of BthMP on A549 cells, concentrating on its impact on cell viability, metastatic processes (migration, invasion, adhesion), reactive oxygen species (ROS) and nitric oxide (NO) production. By comparing its effects on A549 and BEAS-2B cells and assessing its zinc-dependent mechanism, we seek to elucidate the potential of BthMP as a novel therapeutic agent for lung cancer, advancing targeted biotechnological strategies.

## 2. Results

### 2.1. Effects of BthMP on Viability and Proliferation of Normal and Lung Cells

**Figure 1:**
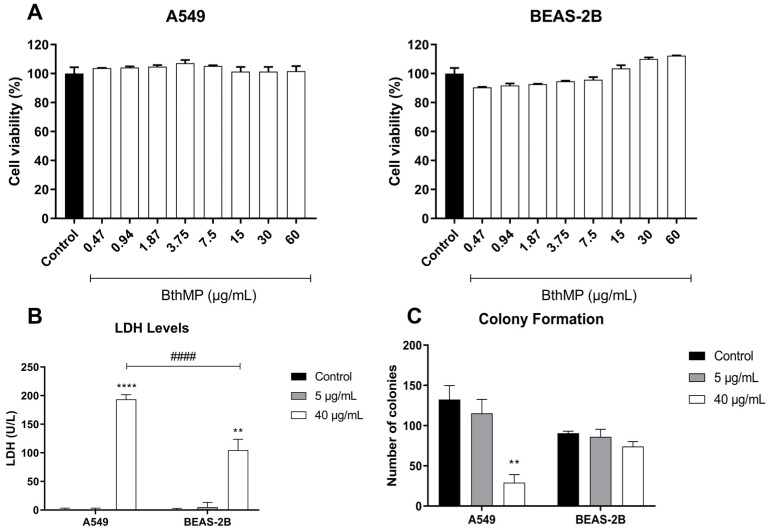
Viability and proliferation of A549 and BEAS-2B exposed to the BthMP treatment for 24 h. (**A**,**B**) Cell viability by MTT assay. Cells incubated only with culture medium had 100% viability. (**B**) LDH assay. (**C**) Colony formation assay. Asterisks (*) indicate a statistically significant difference compared to the control group. ** *p* < 0.01, **** *p* < 0.0001. The hash symbol (#) indicates a statistically significant difference between 40 μg/mL groups. #### *p* < 0.0001.

The effects of BthMP on cell viability and proliferation were assessed in A549 (lung adenocarcinoma) and BEAS-2B (non-cancerous bronchial epithelial) cells using [(3-(4,5-dimethylthiazol-2-yl)-2,5-diphenyltetrazolium bromide)] MTT, Lactate Dehydrogenase (LDH), and colony formation assays. Treatment with BthMP ranging from 60 to 0.47 µg/mL for 24 h did not substantially reduce the metabolic viability of either A549 or BEAS-2B cells compared to the untreated controls (Figure 1A). In contrast, the LDH assay revealed significant cytotoxicity at higher concentrations. Treatment with 40 µg/mL of BthMP significantly increased LDH release in both A549 cells (*p* < 0.0001) and BEAS-2B cells (*p* < 0.01) compared to their respective controls. Notably, the cytotoxic effect was significantly more pronounced in the A549 cancer cells than in the non-cancerous BEAS-2B cells (Figure 1B). At 5 µg/mL, no significant increase in LDH release was observed in either cell line (*p* < 0.0001).

The colony formation assay further demonstrated BthMP’s selective anti-proliferative effect. At 40 µg/mL, BthMP significantly inhibited the ability of A549 cells to form colonies by approximately 75% (*p* < 0.01). However, the same concentration had no significant effect on the colony-forming ability of BEAS-2B cells (Figure 1C). The lower concentration of 5 µg/mL did not significantly impact colony formation in either cell line. These results suggest that while BthMP does not affect short-term metabolic viability, it selectively induces cytotoxicity and inhibits the long-term proliferative potential of A549 cancer cells at 40 µg/mL.

### 2.2. Effects of BthMP on Adhesion, Migration and Invasion of A549 and BEAS-2B

**Figure 2:**
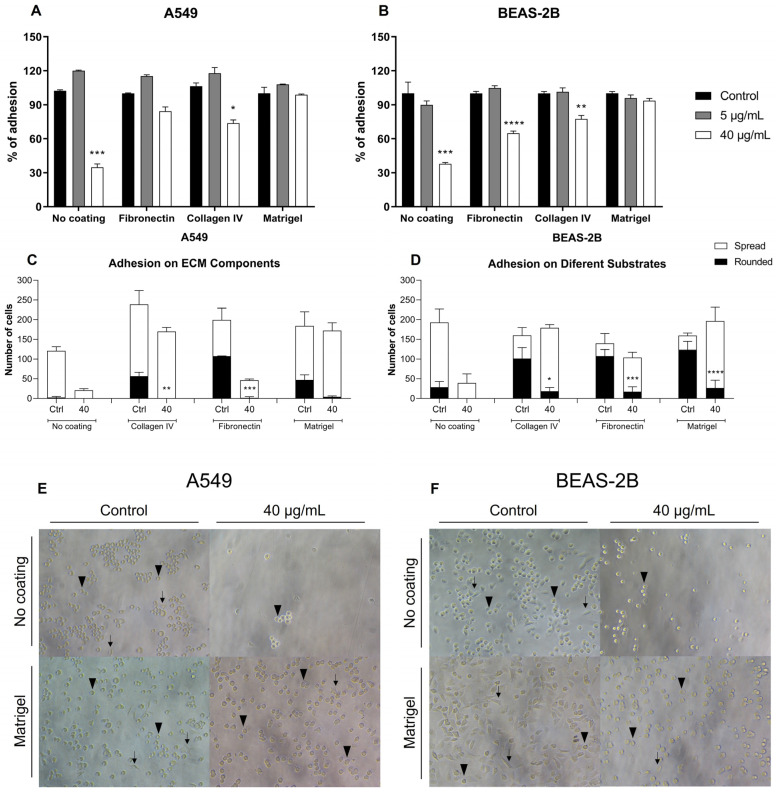
Inhibition of cell adhesion assay on different extracellular matrix components (Collagen IV, fibronectin and Matrigel). A549 and BEAS-2B were pre-treated either with BthMP (5 and 40 μg/mL) or culture medium (negative control) for 30 min. (**A**) Percentage of A549 cell adhesion (%) onto a plate previously coated or not with ECM components. (**B**) Percentage of BEAS-2B cell adhesion (%) onto a plate previously coated or not with ECM components. (**C**) Number of spread and rounded A549s in three extracellular matrix components and with no coating, with BthMP treatment (40 μg/mL). (**D**) Number of spread and rounded BEAS-2Bs in three extracellular matrix components and with no coating, with BthMP treatment (40 μg/mL). (**E**) Representative images of the A549 show differences between the two types of cell morphology. (**F**) Representative images of the BEAS-2B show differences between the two types of cell morphology. Arrow indicates spread cells, and the arrowhead indicates rounded cells—Scale bar: 100 μm. (*) indicates a statistically significant difference compared to the control group. * *p* < 0.05; ** *p* < 0.01; *** *p* < 0.001; **** *p* < 0.0001.

We examined the ability of BthMP to inhibit the cell adhesion of A549 and BEAS-2B cells to various ECM components (i.e., fibronectin, Matrigel, and collagen IV). The results showed that 40 μg/mL of BthMP caused a significant reduction in A549 adhesion to collagen IV (*p* < 0.05) and uncoated plates (*p* < 0.001) (Figure 2A). In BEAS-2B cells, a significant reduction in adhesion to fibronectin (*p* < 0.0001, collagen IV (*p* < 0.01), and uncoated plates (*p* < 0.001) was The explanation was added.also observed with 40 μg/mL of BthMP (Figure 2B). However, a difference was observed between the cell lines, with treatment with BthMP eliciting different responses in cell morphology. The tumor cell A549 was significantly more impaired in morphology than the non-tumor cell BEAS-2B. After treatment, A549 cells showed a drastic decrease in the number of adherent cells to collagen IV *p* < 0.01) and fibronectin (*p* < 0.001), especially those with a spreading morphology, resulting in a population of predominantly round cells that formed agglomerations (Figure 2C). In contrast, although the total number of adherent BEAS-2B cells was also reduced by treatment, a greater number of cells maintained their spreading shape, while fewer round cells were observed (Figure 2D). Representative images visually confirm these effects of BthMP on cell morphology. They show increased rounding and clumping of A549 cells (Figure 2E), while many BEAS-2B cells remained dispersed (Figure 2F).

**Figure 3:**
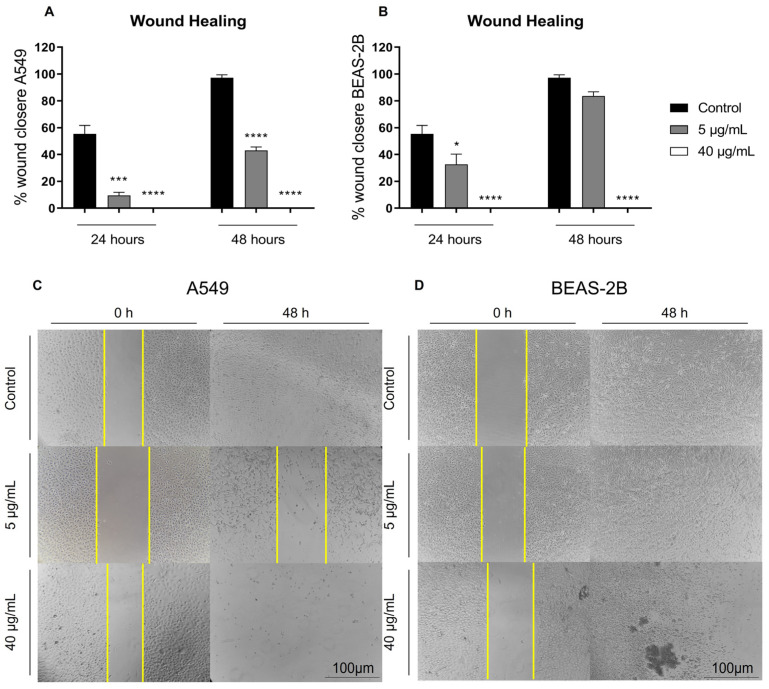
Cell migration by the wound-healing assay. A549 and BEAS-2B were treated with different concentrations of BthMP (5 and 40 μg/mL) or medium (negative control). (**A**) Percentage of wound closure in A549 cells after 24 and 48 h treatment with 5 and 40 μg/mL of BthMP, respectively; (**B**) Percentage of wound closure in BEAS-2B cells after 24 and 48 h in treatment with 5 and 40 μg/mL of BthMP, respectively; (**C**) Representative images of wound closure after 24 and 48 h of A549s upon treatment with BthMP 5 and 40 μg/mL; (**D**) Representative images of wound closure after 24 and 48 h of BEAS-2Bs upon treatment with BthMP 5 and 40 μg/mL. The yellow lines delineate the area where the wound was performed. Scale bar: 100 μm. (*) indicates a statistically significant difference when compared to the control group. * *p* < 0.05; *** *p* < 0.001; **** *p* < 0.0001.

Next, we evaluated the effects of BthMP on horizontal cell migration using the wound-healing assay. In untreated control groups, both A549 and BEAS-2B cells showed robust migratory capacity, achieving nearly complete wound closure by 48 h. Treatment with BthMP resulted in a potent, dose-dependent inhibition of cell migration in both cell lines. In A549 cancer cells, even the low concentration of 5 µg/mL significantly inhibited migration, with wound closure reaching only 9% at 24 h and 43% at 48 h (*p* < 0.0001) (Figure 3A). The higher concentration of 40 µg/mL completely abrogated the migratory ability of A549 cells at both time points. Similarly, in the non-cancerous BEAS-2B cells, BthMP inhibited migration at 24 h, where 5 µg/mL led to a significant but less severe reduction in wound closure (32%, *p* < 0.05). By 48 h, however, the cells treated with 5 µg/mL had largely recovered, achieving 83% closure, a level not statistically different from the untreated control. However, 40 µg/mL also completely arrested migration in BEAS-2B cells (*p* < 0.0001) (Figure 3B).

The representative images visually confirm these results (Figure 3C,D). Notably, the profound inhibition observed at 40 µg/mL in both cell lines appears to be a direct consequence of cytotoxicity and cell detachment, as evidenced by the sparse monolayers and the absence of migrating cells at the wound edge. This suggests that at high concentrations, BthMP prevents wound closure by compromising cell viability and adhesion rather than solely inhibiting the migratory machinery.

**Figure 4:**
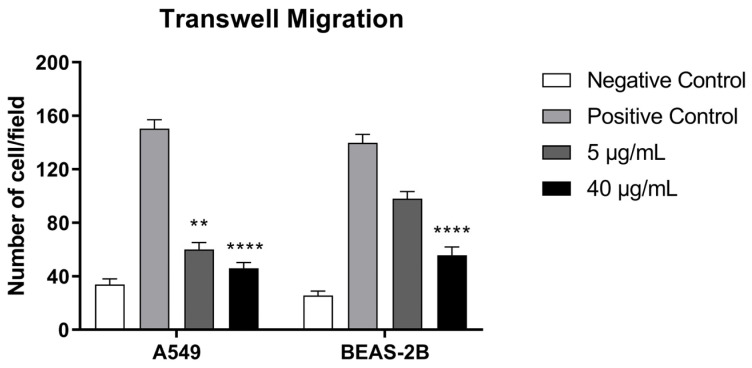
Cell migration by Transwell. A549 and BEAS-2B were pre-incubated with different concentrations of BthMP (5 and 40 μg/mL) or medium (negative control) for 1 h. Number of A549 and BEAS-2B cells migrated per field. (*) indicates a statistically significant difference when compared to the control group. ** *p* < 0.01; **** *p* < 0.0001.

Vertical migration was assessed using a transwell assay to further quantify the effects of BthMP. The results demonstrated a potent and selective inhibition of A549 cancer cell migration. Compared to the positive control, BthMP at concentrations of 5 and 40 µg/mL significantly reduced A549 migration by approximately 60% (*p* < 0.01) and 70% (*p* < 0.0001), respectively (Figure 4).

In contrast, the non-cancerous BEAS-2B cells were notably more resistant. The 5 µg/mL did not cause a statistically significant reduction in migration. A significant inhibition of approximately 60% (*p* < 0.0001) was observed only at 40 µg/mL, which reduced migration to a level comparable to the negative control. These data confirm that BthMP selectively inhibits lung cancer cell migration at lower, non-toxic concentrations.

**Figure 5:**
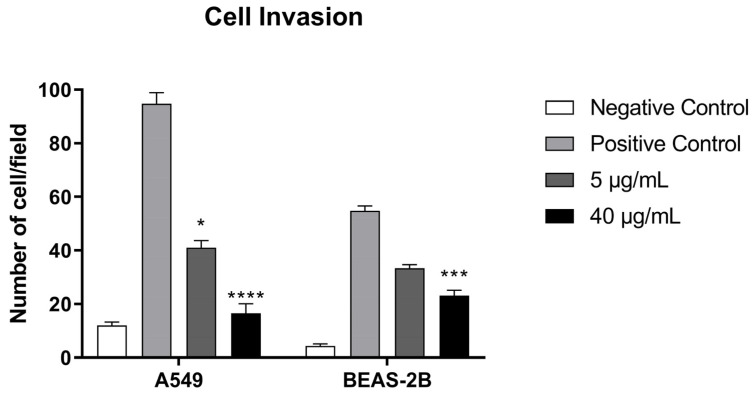
Inhibition of cell invasion by the transwell invasion. Cells A549 and BEAS-2B were pre-incubated with different concentrations of BthMP (5 and 40 μg/mL) or culture medium for 1 h. Number of A549 and BEAS-2B cells invaded per field. (*) indicates a significant difference when compared to the control group. * *p* < 0.05; *** *p* < 0.001; **** *p* < 0.0001.

The effect of svMP on the invasive potential of A549 and BEAS-2B cells was evaluated using a transwell invasion assay. With A549 lung cancer cells, BthMP demonstrated a potent and dose-dependent inhibition of cell invasion through a Matrigel barrier. Compared to the robust invasion seen in the positive control, treatment with 5 µg/mL BthMP significantly reduced the number of invasive cells by approximately 57% (*p* < 0.05) (Figure 5). The higher concentration of 40 µg/mL resulted in an even more profound inhibition, reducing invasion by approximately 83% (*p* < 0.0001) to a level nearing that of the negative control. In contrast, the non-cancerous BEAS-2B cells exhibited less invasive potential overall and were more resistant to BthMP’s effects. At 5 µg/mL, there was no statistically significant reduction in cell invasion compared to the positive control. A significant inhibitory effect was only observed at 40 µg/mL, which reduced BEAS-2B cell invasion by approximately 53% (*p* < 0.001).

**Figure 6:**
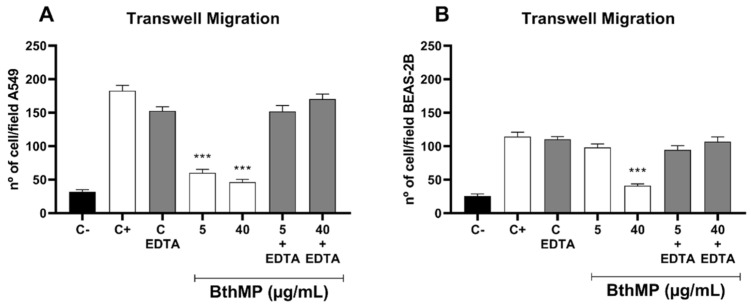
BthMP effect after inactivation by EDTA on A549 and BEAS-2B migration by transwell. A549 and BEAS-2B were pre-incubated with different concentrations of BthMP (5 and 40 μg/mL) or medium (negative control) or BthMP inactivated by EDTA 1 μg/mL for 1 h. (**A**) Number of A549s migrated per field. (**B**) Number of BEAS-2Bs migrated per field C- (Negative Control), C+ (Positive Control) and C EDTA (EDTA Control). (*) indicates a significant difference when compared to the control group. *** *p* < 0.001.

To confirm that the anti-migratory effects of BthMP are mediated by its enzymatic activity, we used ethylenediaminetetraacetic acid (EDTA), a zinc-chelating agent, to inactivate the metalloproteinase. The results clearly show that pre-incubation with EDTA completely abolished the anti-migratory effect of BthMP on both A549 and BEAS-2B cells, restoring migration to control levels (Figure 6). This unequivocally demonstrates that the proteolytic function of BthMP is essential for its activity. This finding is critical because, as a P-I class svMP, BthMP’s biological effects are expected to be driven primarily by its single catalytic domain.

### 2.3. BthMP Increases ROS and NO in A549 Cells, While Reducing at BEAS-2B

**Figure 7:**
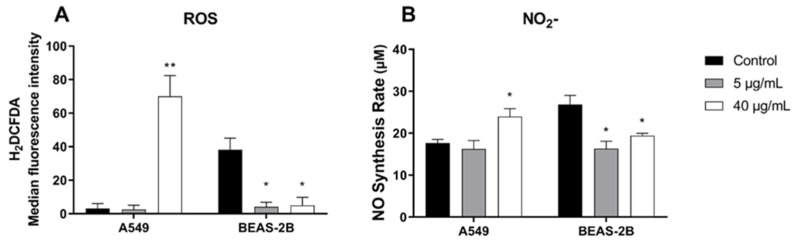
BthMP effect on the production of ROS and NO. (**A**) ROS levels. Cells A549 and BEAS-2B were treated with BthMP (5 and 40 μg/mL). The results were presented as fluorescence intensity; (**B**) Nitrite dosage. Cells A549 and BEAS-2B were treated with BthMP (5 and 40 μg/mL). The NO content was quantified based on the final concentration of the final metabolite nitrite using the Griess assay. (*) indicates a significant difference when compared to the control group. * *p* < 0.05; ** *p* < 0.01.

To assess the impact of BthMP on cellular redox balance, the intracellular ROS levels were quantified using the H2DCF-DA assay, and nitric oxide (NO) was measured using the Griess assay in both A549 and BEAS-2B cells (Figure 7). The two cell lines exhibited markedly different responses. In the A549 cancer cells, treatment with 40 µg/mL BthMP induced a dramatic increase in both ROS and NO production. Specifically, ROS levels increased by approximately 2180% (*p* < 0.01), while NO levels rose by approximately 35% (*p* < 0.05) compared to the untreated controls. The 5 µg/mL did not cause a significant change in either ROS or NO in this cell line (Figure 7A,B). In stark contrast, BthMP treatment led to a significant reduction in these reactive species in the non-cancerous BEAS-2B cells. Both 5 and 40 µg/mL concentrations significantly reduced ROS production by approximately 89% and 87%, respectively (*p* < 0.05). Similarly, NO levels were significantly decreased by approximately 39% and 28% at 5 and 40 µg/mL, respectively (*p* < 0.05). Collectively, these results demonstrate that BthMP exerts opposing, cell-type-specific effects on oxidative and nitrosative stress pathways, increasing the accumulation of reactive species in cancer cells while suppressing it in their non-cancerous counterparts.

## 3. Discussion

This study was designed to evaluate the potential of the P-I metalloproteinase BthMP as a selective anti-cancer agent against lung cancer. Our results demonstrate that BthMP exerts profound anti-cancer effects on A549 lung adenocarcinoma cells while sparing non-cancerous BEAS-2B cells. The observation that BthMP did not reduce cell viability in the short-term MTT assay, even at high concentrations, aligns with prior findings where BthMP showed low acute toxicity on human umbilical vein endothelial cells (HUVEC) [9]. This suggests that BthMP’s primary anti-cancer mechanism is not immediate, widespread cytotoxicity but rather a more targeted mode of action. This selective activity becomes evident in the functional assays. The significant increase in LDH release from A549 cells at 40 µg/mL, which was markedly higher than in BEAS-2B cells, points to a preferential cytotoxic effect involving the disruption of cancer cell membrane integrity. This tumor-selective cytotoxicity is a desirable characteristic for a therapeutic candidate and has been observed with other svMPs, such as Bothropoidin, which also showed greater cytotoxicity against breast cancer cells compared to a normal cell line [11].

Furthermore, the most compelling evidence of BthMP’s selective anti-cancer activity is its potent inhibition of A549 colony formation, a key measure of a cell’s long-term proliferative and tumorigenic potential. BthMP drastically reduced the clonogenic survival of A549 cells without affecting BEAS-2B cells. This specific inhibition of cancer cell proliferation is consistent with the effects of other svMPs, such as jararhagin, which was shown to decrease the proliferation rate of melanoma cells [7]. The mechanism likely involves BthMP’s zinc-dependent catalytic activity targeting key proteins in the tumor microenvironment. As a P-I class svMP, BthMP’s structure is simpler than that of P-III svMPs like jararhagin, which often contributes to lower systemic hemorrhagic activity and thus a more favorable safety profile for therapeutic development. The concentrations used in this study (5 and 40 µg/mL) were effective at revealing a dose-dependent, selective effect at 40 µg/mL, consistent with concentrations used in previous functional studies [9,11]. While these in vitro results are promising, future in vivo studies are essential to validate the efficacy and safety of BthMP as a potential therapeutic agent for lung cancer.

The diverse anti-cancer effects of BthMP can be understood as a cascade of events originating from its primary chemical action: zinc-dependent peptide bond hydrolysis. Our results, confirming that BthMP’s anti-metastatic potential is completely abolished by the zinc-chelating agent EDTA, are crucial as they establish this catalytic function as the essential trigger for all subsequent biological outcomes.

The immediate biological consequence of this enzymatic activity is a profound disruption of cell–matrix interactions. The ability of BthMP to inhibit the adhesion of A549 cells to ECM components is the direct result of this proteolysis. This is consistent with findings for other svMPs that disrupt crucial cell–matrix interactions, such as jararhagin and BpMP-II [12]. The observed morphological changes, particularly the prominent cell rounding and agglomeration in A549 cells, are the visible manifestation of this loss of adhesion [13]. This disruption of cytoskeletal organization is a well-documented effect of svMP action and represents the first physical outcome of BthMP’s primary chemical catalysis.

A direct downstream functional consequence of this compromised adhesion is the potent inhibition of cell migration. Because cells require stable adhesive footholds to generate traction for movement, BthMP’s enzymatic destruction of these connections effectively immobilizes them. Our data from both wound-healing and transwell assays consistently show a dose-dependent inhibition of migration, with remarkable selectivity for A549 cancer cells at lower, non-cytotoxic concentrations. This anti-migratory activity aligns with the known functions of other svMPs, such as Bothropoidin [11], confirming that BthMP can specifically disrupt the migratory machinery of cancer cells by interfering with cell–matrix interactions [14]. At high concentrations (40 µg/mL), the potent blockade of migration is visibly linked to the profound cytotoxicity and cell detachment, which is further explained by the induction of oxidative stress.

This anti-metastatic action is even more pronounced in the context of cell invasion. The ability of BthMP to potently inhibit the invasion of A549 lung cancer cells is a critical finding, as the capacity to invade through the basement membrane is a prerequisite for metastasis [15]. Our results show that BthMP is a powerful inhibitor of this process, particularly for cancer cells. This anti-invasive property is consistent with the activities of other svMPs. For instance, Corrêa et al., 2002 found that the P-III svMP jararhagin inhibited the invasion of SK-MEL-28 melanoma cells by approximately 23–29% [16]. In our study, BthMP demonstrated a substantially more potent effect, achieving an 83% reduction in A549 invasion at 40 µg/mL. This suggests that BthMP may be a particularly effective anti-invasive agent. The profound inhibition of invasion observed in our study is likely a synergistic effect resulting from the multiple activities of BthMP that we have documented. The invasion assay requires cells not only to migrate but also to actively degrade the components of the Matrigel barrier, which mimics the natural extracellular matrix. BthMP attacks this process through a dual mechanism. First, it inhibits cell motility, as demonstrated in our migration assays. Second, it directly degrades the matrix barrier. Our invasion assay provides powerful, albeit indirect, evidence for this proteolytic action. The profound 83% reduction in invasion through the Matrigel barrier at 40 µg/mL cannot be explained by anti-migratory effects alone; it strongly indicates that BthMP is proteolytically dismantling the ECM components of the Matrigel itself, destroying the physical scaffold that the cells must invade.

While direct biochemical verification using methods like gelatin zymography was not performed in this study, this potent activity is consistent with the established proteolytic nature of P-I metalloproteinases. Indeed, identifying the specific ECM substrate profile for BthMP is a critical next step and a clear direction for our future research. Nevertheless, the functional data presented here strongly support the conclusion that BthMP disrupts the metastatic cascade by directly destroying the matrix barrier in addition to inhibiting cell motility. This dual action is compounded by BthMP’s potent anti-adhesive properties, which prevent the cells from gaining the necessary traction to pull themselves through the matrix. The metastatic cascade is a complex series of steps including local invasion, adhesion, and migration [15]. Our data strongly suggest that BthMP disrupts this cascade at multiple critical points, making it a highly effective inhibitor of the invasive process. The greater efficacy against A549 cancer cells, especially at 5 µg/mL, further underscores its potential as a targeted anti-metastatic therapeutic.

Our results align with studies of other P-I svMPs, such as *Bothrops pauloensis* metalloproteinase-II (BpMP-II) from *Bothrops pauloensis*, whose anti-angiogenic effects are also attributed to its proteolytic mechanism [13]. This catalytic-dependent mechanism contrasts with that of more complex P-III svMPs, such as Bothropoidin, which possess additional domains (e.g., disintegrin-like) that can directly bind to cell surface integrins to inhibit adhesion and migration [17]. Therefore, it is highly likely that BthMP exerts its anti-adhesive, anti-migratory, and anti-invasive effects by proteolytically degrading key protein components of the extracellular matrix. By cleaving these substrates, BthMP disrupts the critical cell–matrix interactions that are essential for cell adhesion and movement [18,19]. In essence, our results confirm that the catalytic activity of BthMP is the primary driver of its observed anti-metastatic potential.

Finally, beyond these physical anti-metastatic effects, the BthMP-induced loss of adhesion triggers a potent downstream intracellular signaling consequence that culminates in selective cytotoxicity. Our study reveals that BthMP dramatically and differentially alters the redox environment in lung epithelial cells, inducing a massive surge in ROS and NO in A549 cancer cells while suppressing them in non-cancerous BEAS-2B cells. This opposing effect is a critical finding, suggesting a mechanism for BthMP’s selective cytotoxicity. It is important to clarify that this induction of oxidative stress is not a direct catalytic action of the enzyme, but rather a secondary cellular response. We hypothesize that BthMP’s primary proteolytic activity against extracellular matrix or cell-surface proteins disrupts cell adhesion. This loss of anchorage is a well-documented trigger for anoikis, a cell death program often initiated by a massive burst of intracellular ROS from sources such as mitochondria and NADPH oxidases. Thus, the zinc-dependent catalytic activity of BthMP acts as the upstream trigger for a downstream signaling cascade within the cancer cell that culminates in lethal oxidative stress. In the A549 cells, the substantial increase in both ROS and NO likely pushes the cells into a state of severe oxidative and nitrosative stress, which is known to be cytotoxic. While low to moderate levels of ROS can promote cancer cell proliferation and migration [20,21], the massive 2180% increase we observed likely surpasses a tolerable threshold, triggering cell death pathways. This is consistent with findings by Brown, 2019, who demonstrated that ROS production was essential for the apoptosis induced by *Crotalus atrox* venom [22]. The concurrent increase in NO is also significant. High levels of NO are pro-apoptotic and, when combined with high ROS, can lead to the formation of highly reactive peroxynitrite [23]. This is particularly relevant in the context of lung cancer, where elevated nitrative stress is a known hallmark [24]. Peroxynitrite can damage cellular components and inactivate crucial tumor suppressors like p53, further contributing to the toxin’s anti-cancer effect [25]. The observation that another P-I svMP, *Bothrops pirajai* metalloproteinase (BpirMP), also increases NO levels in vivo provides a precedent for this activity [26].

The inhibition of cell adhesion observed in A549 cells may also be related to the rapid increase in ROS levels. Li et al., 1999, demonstrated that endothelial cell detachment results in a rapid and dramatic increase in intracellular ROS levels; ROS appear to be important in modulating endothelial cell death; and anoikis occurs through an oxidation-sensitive pathway [27].

In stark contrast, treatment with BthMP led to a significant reduction in basal ROS and NO levels in non-cancerous BEAS-2B cells. This suggests a possible protective or redox-buffering effect in normal cells, a mechanism that remains to be explored. This differential response is the most compelling aspect of our results. The ability of a single compound to simultaneously induce a high-stress pro-apoptotic state in cancer cells while attenuating oxidative stress in non-cancer cells is a highly desirable property for a therapeutic agent. This selectivity could lead to a broader therapeutic window and less off-target toxicities, emphasizing the potential of BthMP as a framework for the development of new, targeted anti-cancer drugs.

Although this study provides new and convincing insights into the selective anti-cancer potential of BthMP, several limitations must be acknowledged. First and foremost, this study was conducted exclusively in vitro. Although essential for elucidating cellular mechanisms and proof of concept, two-dimensional cell culture models cannot fully replicate the complex, three-dimensional architecture and physiological conditions of a tumor in a living organism. Factors such as systemic pharmacokinetics, metabolism, host immune response, and the complex interplay within the tumor microenvironment were not considered. Therefore, the promising effects observed here need to be validated in appropriate in vivo animal models of lung cancer to evaluate efficacy, toxicity and therapeutic potential in a physiological context. Secondly, the study relied on two specific cell lines: A549 as a model for adenocarcinoma of the lung and the immortalized BEAS-2B line as a non-cancerous control. Lung cancer is a very heterogeneous disease, and these cell lines may not represent the full spectrum of its subtypes (e.g., squamous cell carcinoma, small cell lung cancer). Even though an immortalized cell line such as BEAS-2B is a standard model, it may not perfectly reflect the response of primary, healthy human bronchial epithelial cells. Future studies should include a broader range of cancer cell lines and, if possible, primary cells to confirm the selectivity of BthMP effects. Finally, while our results clearly point to specific mechanisms of action, further studies are needed to fully characterize the molecular pathways. For example, we have shown that the catalytic activity of BthMP is essential for its function, but the specific extracellular matrix proteins that are cleaved as primary targets have not been identified. Although we observed a dramatic and differential effect on ROS and NO production, we could not determine the exact signaling cascade leading to this redox imbalance and its direct causal link to cell death. Future research using proteomic approaches to identify substrates and experiments on rescue by antioxidants would provide more clarity on the mechanism. Despite these limitations, this study provides a solid foundation and a clear rationale for advancing the investigation of BthMP as a promising candidate for the development of new antimetastatic therapeutics for lung cancer.

## 4. Materials and Methods

### 4.1. Cell Culture

Human lung adenocarcinoma (A549) and non-cancerous bronchial epithelial cells (BEAS-2B) were acquired from the American Type Culture Collection (ATCC, Manassas, VA, USA; A549: ATCC^®^; BEAS-2B: ATCC^®^). Cells were maintained in Roswell Park Memorial Institute (RPMI) 1640 medium (Sigma, São Paulo, Brazil) supplemented with 10% fetal bovine serum (FBS; Cultilab, Campinas, Brazil), 2 mM L-glutamine, 2 mM sodium pyruvate, 1 mM non-essential amino acids, 100 U/mL penicillin and 100 µg/mL streptomycin (all Sigma, São Paulo, Brazil). Cultures were maintained at 37 °C with 5% CO_2_.

### 4.2. BthMP Purification

BthMP was purified from *Bothrops moojeni* venom (obtained from the Herpetology Laboratory from Butantan Institute, batch # 01/16-2) as described by Gomes et al., 2009 [10] with modifications [9]. The molecular mass, purity, and zinc-dependent metalloproteinase activity of BthMP have been previously characterized, confirming it as a P-I class metalloproteinase with a molecular weight of approximately 23 kDa [10]. Our recent work has also confirmed the identity and purity of the enzyme used in our functional assays using mass spectrometry [9]. Briefly, venom was fractionated using a Mono Q™ (5/50 mm) (Cytiva, São Paulo, Brazil) strong anion-exchange column, equilibrated with 50 mM ammonium bicarbonate buffer (AMBIC), pH 7.8 and eluted with 50 mM to 500 mM AMBIC gradient. Active fractions were pooled and further purified on HiTrap CM Sepharose FF weak cation-exchange column (Cytiva, São Paulo, Brazil) (1 mL) equilibrated with a 50 mM AMBIC and eluted with 50 mM to 500 mM AMBIC gradient. The fractions of interest were pooled, lyophilized, suspended in AMBIC 50 mM and applied to HiPrep Sephacryl S-200 HR (16/60) gel filtration column (Cytiva, São Paulo, Brazil). Purity was verified by 12% sodium dodecyl sulfate-polyacrylamide gel electrophoresis (SDS-PAGE), confirming a single ~23 kilodaltons (kDa) band and identity was confirmed by mass spectrometry (LTQ-Orbitrap Velos ETD, Thermo Scientific, Waltham, MA, USA). All purification steps were performed using columns from Cytiva and an ÄKTA Pure system (Marlborough, MA, USA).

### 4.3. Evaluation of Cytotoxicity by MTT Assay

A549 and BEAS-2B cells (3 × 10^4^ cells/well) were seeded in 96-well microplates and incubated for 24 h at 37 °C and 5% CO_2_ in RPMI 1640 medium. Viability and cell count were determined using a Neubauer chamber and 0.4% trypan blue staining [28]. Cells were treated with twofold serial dilution of BthMP (ranging from 60 to 0.47 µg/mL) or culture medium (negative control) for 24 h. The volume of 10 µL/well of MTT (3-(4,5-Dimethylthiazol-2-yl)-2,5-Diphenyltetrazolium Bromide–Sigma, São Paulo, Brazil solution (5 mg/mL in 1× PBS) was added to each well and incubated for 3 h at 37 °C with 5% CO_2_. Formazan crystals were dissolved in 100 µL 1× PBS containing 10% sodium dodecyl sulfate (SDS) and 0.01 M HCl for 18 h at 37 °C and 5% CO_2_. Absorbance was measured at 570 nm using a Multiskan GO plate reader (Thermo Scientific, Waltham, MA, USA). Cell growth inhibition (%) was calculated as [1 − (OD treated/OD control)] × 100.

### 4.4. Colony Formation Assay

The colony formation assay was made according to the previous method with modifications [29]. Briefly, A549 and BEAS-2B cell lines (3 × 10^4^ cells/well) were seeded in 96-well microplates with complete RPMI-1640 medium and incubated at 37 °C and 5% CO_2_ for 24 h. They were then treated with BthMP at concentrations of 5 and 40 μg/mL or culture medium (control group) for 24 h. After treatment, the cells were harvested and seeded in 6-well plates (100 cells/2000 μL/well) for two weeks at 37 °C and 5% CO_2_. The culture medium was then removed, the cells were washed with 1× PBS, fixed with 100% cold methanol for 10 min, and then stained with Panotic Kit (Laborclin, Pinhais, Brazil). The colonies developed by more than 50 cells were computed and photographed. Three fields per well were photographed using a Nikon Eclipse TS100 microscope (Thermo Scientific, Waltham, MA, USA) (10× magnification).

### 4.5. LDH Assay

The LDH release assay was carried out following the method described by Legrand et al. 1992 [30]. Briefly, A549 and BEAS-2B cells were seeded at 3 × 10^4^ cells per well in 96-well microplates. After 24 h, cells were treated with BthMP (5 and 40 μg/mL) or culture medium (control) and incubated at 37 °C and 5% CO_2_ for 24 h. The culture supernatant was then collected and subjected to LDH measurement according to the instructions of the LDH UV Lactic Dehydrogenase Kit (Bioclin, Belo Horizonte, Brazil).

### 4.6. Cell Adhesion Inhibition Assay

To determine the interactions between BthMP and extracellular matrix components, collagen IV (10 μg/mL in 0.1 M acetic acid), fibronectin (5 μg/mL in 1× PBS), or Matrigel™ (1:10, Corning ^®^, Union City, CA, USA) was applied to 96-well microplates and incubated at 4 °C for 18 h. A control without any extracellular matrix coat was performed. The wells were carefully washed twice with 1× PBS and blocked with bovine serum albumin (BSA) in PBS (0.1%, 50 μL). A549 and BEAS-2B cells (3 × 10^4^ cells/well) were pre-incubated with BthMP (5 and 40 μg/mL) or medium (control group) for 1 h at 37 °C. Cells were then seeded and incubated for 2 h at 37 °C and 5% CO_2_. Detached cells were removed by washing with 1× PBS, and adherent cells were quantified by MTT assay as described previously.

### 4.7. Wound-Healing Assay

Cell migration was quantified using a modified wound healing assay previously described by Gimenes et al., 2017 [17]. A549 and BEAS-2B cells (1 × 10^6^ cells/well) were seeded in 12-well plates with complete RPMI 1640 medium and incubated at 37 °C and 5% CO_2_ for 24 h. The medium was then removed, and the cell monolayers were scored with a 200 μL plastic pipette tip. The medium was then replaced, and cells were treated with BthMP toxin (5 and 40 μg/mL) or medium (control) for 24 and 48 h. Images were captured with an inverted phase contrast microscope (Nikon Eclipse TS100, Thermo Scientific, Waltham, MA, USA) immediately after scribing (t = 0 h) and after 24 or 48 h of treatment (t = 0 h). Cell migration was calculated using Image J software (v.1.54d) and expressed as a percentage of wound closure:

% wound closure = [(At = 0h − At = 0h)/At = 0h] × 100

where “At = 0h” h is the wound area measured after scratching and “At = 0h” is the wound area measured 24 or 48 h after scratching.

### 4.8. Cell Migration and Invasion Assays

A transwell chamber (Corning^®^, Union City, CA, USA) with an 8 μm membrane was used to assess vertical cell migration and invasion according to [9,17]. For the migration assay, A549 and BEAS-2B were pre-treated with BthMP (5 and 40 μg/mL) for 1 h at 37 °C and 5% CO_2_ in RPMI 1640-FBS-free culture medium or with BthMP (5 and 40 μg/mL) inactivated by EDTA (1 mM) for 1 h at 37 °C and 5% CO_2_ in RPMI 1640-FBS-free medium. After incubation, 7 × 10^4^ cells were seeded in the upper part of the insert chamber, and medium containing 10% FBS was added to the lower compartment as a chemoattractant for the cells. In the negative control, the lower layer was filled with a culture medium without FBS.

For the cell invasion assay, the upper chamber of the transwell plates was earlier coated with Matrigel (Corning^®^ Matrigel^®^ Matrix, Union City, CA, USA) and a 1:10 dilution (Matrigel/PBS) with serum-free medium for 30 min before the cells were seeded. The cells were pre-incubated as described previously and placed in the upper chamber of the Transwell. After 24 h, the migrated or invaded cells were stained with Panotic Kit (Laborclin, Pinhais, Brazil), and determined by counting the cells in 6 fields, which were randomly examined under a microscope (Nikon Eclipse TS100, Thermo Scientific, Waltham, MA, USA) at 10× magnification.

### 4.9. Production of Nitric Oxide

A549 and BEAS-2B (3 × 10^4^ cells/well) were seeded in 96-well microplates, and after 24 h, BthMP (5 and 40 μg/mL) or medium (control) was added to the cultures and incubated at 37 °C and 5% CO_2_. After 24 h, the supernatant was collected for quantification of NO. The NO content was quantified based on the final concentration of the final metabolite nitrite using the Griess assay according to a previously published protocol [31]. The 5 μL aliquots of test solution (standard, control, experimental sample) were added to 0.1% Griess reagent solution (50 μL 3.9 mM N-(1-naphthyl) ethylenediamine in 5% (*v*/*v*) phosphoric acid) and incubated for 10 min in the dark at room temperature. Sulfanilamide solution (1% in phosphoric acid) was added to the mixture, and the absorbance was measured at 540 nm using a microplate reader. A standard solution of sodium nitrate (0.1 mM) was used to prepare a standard curve to determine the actual concentrations of the samples. Cell-free controls were included to ensure BthMP did not interfere with the Griess assay, and results were normalized to untreated cells.

### 4.10. ROS Production

ROS production in A549 and BEAS-2B was evaluated by intracellular peroxide-dependent oxidation of 2′,7′-dichlorodihydrofluorescein (H_2_DCF-DA) (Invitrogen, Carlsbad, CA, USA, catalog number: D399) to form the fluorescent compound 2′,7′-dichlorofluorescein (DCF) as previously described by Santos et al., 2024 [32]. A549 and BEAS-2B cells were placed in dark 96-well microplates with 3 × 10^4^ cells per well. After 24 h, new medium with BthMP (5 and 40 μg/mL) or medium (control) with the carrier was added and incubated at 37 °C and 5% CO_2_ for 24 h. After treatment, cells were washed with 1× PBS and incubated with 150 μL H_2_DCF-DA (10 μM; diluted in 1 PBS with 10% FBS) for 45 min at 37 °C and 5% CO_2_ in the dark. DCF fluorescence intensity was immediately measured using a GloMax Explorer Multiwell Scanning Spectrophotometer (Promega, Madison, WI, USA). The results were presented as fluorescence intensity. Hydrogen peroxide (H_2_O_2_) was used as a positive control for ROS production, and the negative control corresponds to cells treated with culture medium only. Control wells containing only culture medium with BthMP (cell-free) were included to account for any potential auto-oxidation or fluorescence interference; these showed a negligible background signal.

### 4.11. Statistical Analysis

The results were obtained from three independent experiments, each with three technical replicates, and expressed as mean ± standard error of the mean (SEM). Normality was tested using the Shapiro–Wilk test. Parametric data were analyzed with Student’s *t*-test or one-way analysis of variance (ANOVA) followed by Tukey’s post-test. Non-parametric data were analyzed with Mann–Whitney or Kruskal–Wallis tests followed by Dunn’s post-test using GraphPad Prism 7.0. Statistical significance was set at *p* < 0.05.

## 5. Conclusions

This study demonstrates for the first time that BthMP, a P-I metalloproteinase from *Bothrops moojeni* venom, exhibits potent and selective anti-cancer effects against A549 lung adenocarcinoma cells. BthMP impaired key metastatic processes like adhesion, migration, invasion and reduced proliferative capacity, effects dependent on its zinc-mediated catalytic activity, as confirmed by inhibition with EDTA. Notably, BthMP also induced oxidative and nitrosative stress in cancer cells, while exerting a contrasting protective effect in non-cancerous BEAS-2B cells. This dual modulation of redox status highlights a selective mechanism of action. In conclusion, BthMP acts through a dual mechanism of matrix proteolysis and redox imbalance, and this selective, dual-pronged mechanism underscores the potential of BthMP as a promising therapeutic scaffold for the development of novel anti-metastatic agents for lung cancer, reinforcing the value of natural toxins as a source for innovative oncology research.

## Data Availability

The original data presented in this study are included in this article. Further inquiries can be directed to the corresponding authors.

## Abbreviations

The following abbreviations are used in this manuscript: NSCLC, non-small cell lung cancer; ECM, extracellular matrix; svMPs, Snake venom metalloproteinases; BthMP, Bothrops metalloproteinase; ROS, reactive oxygen species; NO, nitric oxide; A549, lung adenocarcinoma; BEAS-2B, non-cancerous bronchial epithelial; MTT, [(3-(4,5-dimethylthiazol-2-yl)-2,5-diphenyltetrazolium bromide)]; LDH, Lactate Dehydrogenase; FBS, Fetal bovine serum; EDTA, ethylenediaminetetraacetic acid; PBS, phosphate-buffered saline; HUVEC, human umbilical vein endothelial cells; tEnd, thymus-derived Endothelial cells; TSV-DM, *Trimeresurus stejnegeri* venom-derived metalloproteinase; BpMP-II, *Bothrops pauloensis* metalloproteinase-II; BpirMP, *Bothrops pirajai* metalloproteinase; RPMI, Roswell Park Memorial Institute; AMBIC, ammonium bicarbonate buffer; kDa, kilodaltons; SDS-PAGE, sodium dodecyl sulfate-polyacrylamide gel electrophoresis; SDS, sodium dodecyl sulfate; BSA, bovine serum albumin; H_2_DCF-DA, 2′,7′-dichlorodihydrofluorescein; DCF, 2′,7′-dichlorofluorescein; H_2_O_2_, hydrogen peroxide; SEM, standard error of the mean; ANOVA, analysis of variance.
